# Effects of poling camber angle on the biomechanics of cross-country sit-skiing

**DOI:** 10.1038/s41598-023-48359-z

**Published:** 2023-11-28

**Authors:** Yuan Tian, Xue Chen, Yujie Liu, Gang Sun, Zhixiong Zhou, Chenglin Liu, Bo Huo

**Affiliations:** 1 Sports Biomechanics Center, Sports Artificial Intelligence Institute, Capital University of Physical Education and Sports, No. 11 North Third Ring Road West, Beijing, 100191 People’s Republic of China; 2 Biomechanics Lab, Department of Mechanics, School of Aerospace Engineering, Beijing Institute of Technology, No. 5 South Zhongguancun Street, Beijing, 100081 People’s Republic of China; 3 School of Physical Education and Coaching Science, Capital University of Physical Education and Sports, Beijing, 100191 People’s Republic of China

**Keywords:** Health care, Engineering

## Abstract

Cross-country sit-skiers use double poling (DP) technique to drive the slide. The aim of this study is to analyze how poling camber angle affect the capacity of power output and biomechanical parameters of the DP process. Twenty-four non-disabled college students (24.67 ± 1.46 years old) were recruited to perform three successive 30-s maximal effort tests with different poling camber angles of 0°, 15°, 24° and 30° using a sit-skiing ergometer. The biomechanical parameters, output power and muscle activation of the subjects were analyzed. The results showed that DP output power increased with the increase of poling camber angle at 15° (597.78 ± 150.31 J), 24° (610.94 ± 158.96 J,* P* = 0.011) and 30° (629.10 ± 168.78 J, *P* < 0.001) compared with 0° (590.65 ± 148.95 J). However, effective output power decreased with the increase of camber angle. Poling with camber angle of 24° had the shortest cycle time 1.53 ± 0.17 s, compared with other abduction angle (0°, 1.57 ± 0.19 s, 15°, 1.55 ± 0.16 s, and 30°, 1.56 ± 0.19 s). Compared with 0° (1.02 ± 0.14 m), the cycle distance significantly increased at poling camber angles of 24° (1.07 ± 0.12 m, *P* = 0.029) and 30° (1.11 ± 0.13 m, *P* < 0.001). With the increase of poling camber angle, the shoulder and elbow joint range of motions and joint moments were significantly increased. This study found that poling with shoulder abducted increased the output power but decreased the efficiency. By analyzing the poling angle and poling force, we find that the optimal poling camber angle may depend on the terrain or the skiing speed. These results may guide the competition techniques and tactics in the matches, and may further influence the strength-training programs of cross-country sit-skiing athletes.

## Introduction

Cross-country sit-skiing is an aerobic endurance sport in the Winter Paralympics Games. Athletes with amputation, spinal cord injuries, cerebral palsy, and growth defects sit on a sit-ski and generate propulsion with upper limbs by using a pair of poles. The pushing technique performed by sit-skiers is adopted from the double poling (DP) technique used by standing non-disabled skiers. DP refers to a skiing technique in which both poles are planted to the ground simultaneously and trunk flexion is synchronized with shoulder and elbow extension to create propulsive force^1^. The determinants of cross-country skiing usually include aerobic capacity, muscle strength^2^ and technical skill^3^. Accumulated evidences have shown that the upper body power (UBP) has an important role in cross-country ski racing, which was further confirmed by statistical analysis from both aerobic energy systems^4^ and shorter sprint-type UBP tests^5^. For instance, the factors such as one-repetition maximum (1-RM) strength^6^, lean mass^7^, isokinetic muscle strength^8^, maximum oxygen uptake^9^ have been confirmed to be correlated with the skiing or sit-skiing performance. The technical factors such as pole angle^10–12^, pacing length^12–14^, poling phase^12,15^, body inclination^10^, angular velocities of elbow- and hip-flexion^3^ were also found to influence the cross-country skiing.

Seating posture, which is associated with the functional control of athletes’ trunk and hip muscles^16^, has recently been proved to be a key technical factor in cross-country sit-skiing^16–18^. For example, athletes with low trunk control usually use a knee high (KH) posture to gain more support from the seat with knees higher than hip, which exerted less reaction force on the shoulder joint and could reduce the risk of lower back problems^11^. While seated with knees lower than hip (KL) was found to improve respiration, but impede their performance and efficiency^16^.

The above studies in dissecting the poling technique of cross-country sit-skiing meanly focused on the technique in the sagittal plane. During these tests, athletes performed the DP in a standard technique, with the shoulder flexion and then extension to drive the sled. But, it was not always the case in the races, that athletes were observed to pole with camber angle in the Paralympic Games of cross-country sit-skiing^19^. During a competition, athletes attempt to glide with the pole inclined out. With shoulder abducted during the poling, the pole force will be resolved to a vertical, a propulsive and a mediolateral component. The propulsive component is function of the poling camber angle:

$$ F_{{{\text{propulsive}}}} = F_{pole} \times \sin (pole\, angle)\times \cos (poling\, camber\,  angle), $$

which means that the more the shoulder abducts with the greater poling camber angle, the greater the loss of the propulsive force. This is an import factor that determining the efficiency of DP in cross-country sit-skiing, but we hardly found any relevant studies on this issue.

There are similar problems in the wheelchair propulsion process, as we found that the wheels of sports wheelchair are not installed vertically, but have an inclined angle with the ground^20–22^. Mason et al.^23^ found that a wheelchair camber angle of 24° produced greater power than camber angles of 15° and 20°. While another study found that a camber angle of 18° shows a good effect on the maximal effort mobility performance in wheelchair athletes^24^. These studies showed that poling camber angle can affect the propulsion process of wheelchair user.

The purpose of this study is to investigate the effect of poling camber angle on biomechanical parameters during DP propulsion of cross-country sit-skiing. By using a self-designed ergometer, athletes were asked to perform DP with poling camber angle, and evaluate how poling camber angle affect the capacity of power output and biomechanical parameters of cross-country sit-skiing. The results of the study can help to understand the effects of poling camber angle on propulsion mechanics, and may provide instructions on the training of cross-country sit-skiers.

## Materials and methods

### Participants

Twenty-four non-disabled students were recruited from the Capital University of Physical Education and Sports, who are 12 females and 12 males, 24.67 ± 1.46 years old, 168.75 ± 6.78 cm height and 62 ± 10.39 kg in mass. Inclusion criteria were as follows: participants who majored in physical training. The subjects suffering from injuries during the testing period were excluded.

This study was approved by the Ethics Committee of the Capital University of Physical Education and Sports (Beijing, Peoples’ Republic of China), and all experiments were performed in accordance with relevant guidelines and regulations. Before the start of the study, participants were informed about the design of the study, with a special emphasis on the possible risks and benefits, and all participants provided written informed consent prior to the enrolment.

### Physical test

**Figure 1:**
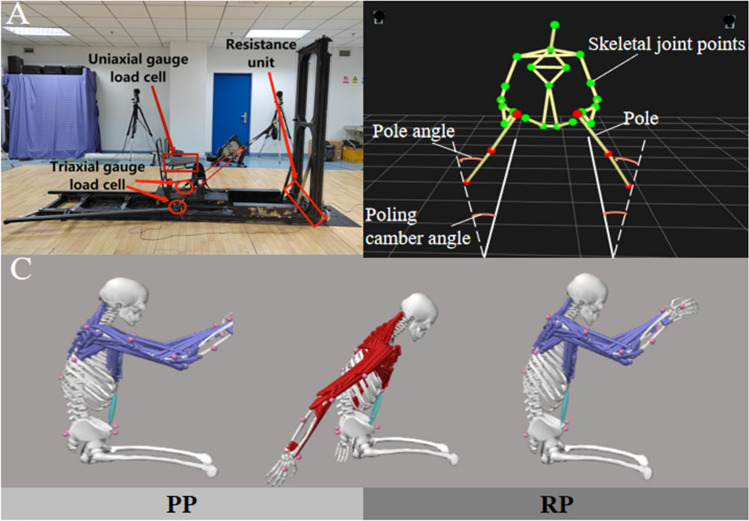
Equipment and the schematic diagram of double poling technique. (**A**) The intelligent training and experimenting equipment of cross-country sit-skiing. (**B**) The schematic diagram of double poling with shoulder abducted. (**C**) Action division in PP and RP.

All tests were performed on the intelligent training and experimenting system of cross-country sit-skiing (Fig. 1A) in the lab environment with a temperature of 23 °C and suitable humidity. Before the formal test, subjects were introduced to the testing equipment and allowed to warm-up at self-selected resistance and poling rhythm for 5 to 10 min. After the warm-up, reflective marker points and surface EMG sensor electrodes were fixed by using strips of adhesive Velcro, and maximum voluntary contraction (MVC) exercises were performed for each muscle to normalize EMG signals.

Subjects were asked to sit with their lower leg strapped to the seat to simulate the conditions of athletes with disabled lower limb, and then performed three successive 30-s maximal effort tests (poling camber angle at 0°, 15°, 24°, 30°) of DP with 3 min rest between each group and the three consecutive tests. The poling resistance was set at 5% of body weight, which was chosen from several pilot testing and gave skiers the most natural feeling of double-poling^8^.

### Equipments and data collection

Physical tests were performed on an intelligent training and experimenting system of cross-country sit-skiing (Fig. 1)^8^. The device can adjust the angle of two rails, which offers non-parallel rails to simulate poling with different camber angles. By using the new system, participants performed DP to simulate the cross-country sit-skiing propulsion process with different poling gestures. The poling forces were recorded at 50 Hz.

Three-dimensional kinematic data were recorded by seven Oqus3 + cameras (Qualisys AB, Gothenburg, Sweden) at 200 Hz. A full-body marker set^25^ was employed and modified, 34 markers, diameter 10 mm, including 2 on each pole, 2 on each guide rail and 28 markers (including the trunk (6), pelvis (4), right femur (2), left femur (2), right humerus (4), right radius (1), right ulna (1), right hand (1), left humerus (4), left radius (1), left ulna (1), and left hand (1)) on body were used in the system. The two markers on each pole were used to determine the force direction of the pole force.

Muscle activation was recorded by using a 16-channel surface electromyography (EMG) device (Yishi KangLian Technology Co., Ltd, Shanghai, China) with a sampling frequency of 1000 Hz and was synchronized to human kinematic. Muscle activity signals of 6 muscles: Anterior deltoid (Antdelt), Middle deltoid (Middelt), Posterior deltoid (Postdelt), Biceps brachii (Bic), Triceps brachii (Tric), and Infraspinatus (Infra) muscles of the right side were recorded. Electrodes were placed with guidance from Surface ElectroMyoGraphy for the Non-Invasive Assessment of Muscles (http://www.seniam.org).

### Data analysis

During the cross-country sit-skiing simulation, a DP cycle is divided into a poling phase (PP) and a return phase (RP). PP was defined with start with the pole tips in its foremost position and ending in their rearmost position; the RP was defined as the opposite. Cycle time (CT), poling time (PT), and recovery time (RT) were the duration of the DP cycle, PP, and RP respectively. All biomechanical variables were calculated from six consecutive stable DP cycles in the middle stage of the poling simulation. The data were collected from the right side of the subject.

Joint angles were defined, in the anatomical position as follows: shoulder = 0° (flexion positive, extension negative), elbow = 0° (flexion positive), spine flexion = 0° (angle between pelvis and trunk in sagittal plane, kyphosis/flexion negative), and pole angle in sagittal plane where horizontal (= 0°) and pole angle vertical (= 90°)^16^. We affixed marker points to the slider and the mean distance of a PP was measured by monitoring the markers sticking on the poling platform using the Qualisys system. The cycle distance is the total distance between the PP and the RP. Output power during the poling cycle was defined as: Output Power = barbell movement distance × barbell gravity, and the barbell movement distance was represented by the marker movement sticking on the poling platform. Effective power was defined as

$$ Effective\, power = \frac{{output\, power}}{{\cos (camber\,angle)}}. $$

By using OpenSim software, individual size and body proportions were used to scale the model. Scaling is typically performed by comparing experimental marker data to virtual markers placed on a model. In addition, the segment masses and inertia properties were scaled from body mass^26^, so that they better match the experimental data. A digital low-pass filter with a cut-off frequency of 13 Hz was used to filter the measured kinematics data. Then the inverse kinematics (IK) was performed to estimate the joint angles of the particular subject from experimental data. Poling reaction force was simulated by a point force applied to the hand. Then, inverse-dynamics equations were solved using kinematic data and external force data. Finally, static optimization (SO) was used to calculate the muscle forces.

EMG signals were integrated by a bandpass filter ranging from 50 to 300 Hz and then processed with full-wave rectification and linearly enveloped, and finally normalized by MVC^16^. For each cycle the average muscle activity (aEMG) and the muscle activity peak (EMGpeak) were calculated on the rectified signals and on the linear envelope respectively^3^.

### Musculoskeletal modeling

We used a bilateral upper extremity trunk model for cross-country sit-skiing double poling propulsion^27^. The cross-country sit-skiing model is developed by combining three previously built OpenSim models. In brief, full-body lumbar spine (FBLS)^28^ was adopted as the base model; the DAS3 model^29^ provides rotator cuff muscles and spanning elbow joint muscles; the human shoulder model^30^ provides the body properties of the scapula and clavicle.

### Statistical analysis

Values were recorded as mean values ± standard deviations (SD). All data are averaged from three tests. Data were checked for normality by using the Shapiro–Wilk analysis. The data were analyzed by one-way ANOVA of repeated measures, with Bonferroni post-test to determine the poling camber angle at which the difference across trials occurred. Statistical significance level was set at P < 0.05. All data were analyzed by using SPSS version 26.0.

## Results

### Temporal–spatial parameters

**Figure 2:**
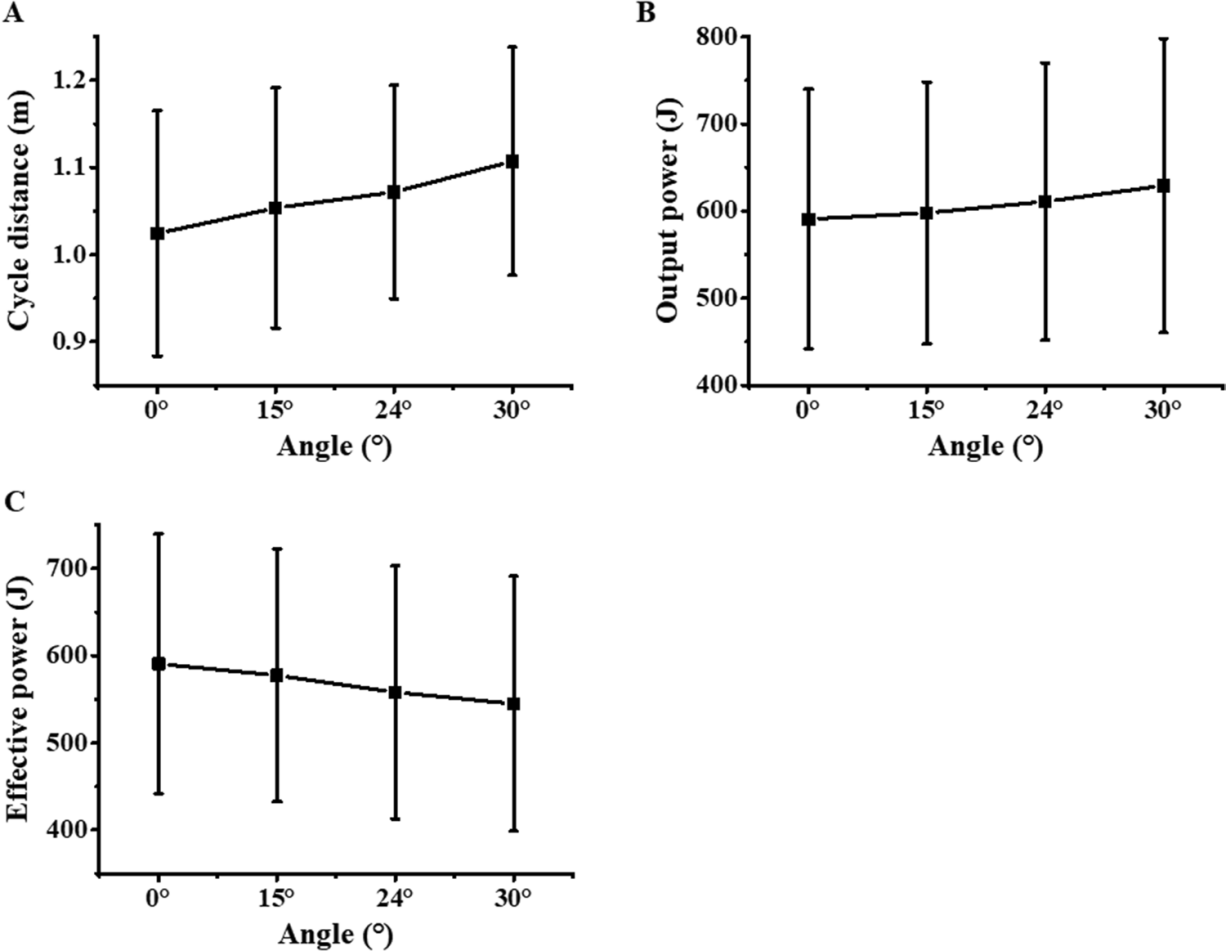
Cycle distance and output power of different poling camber angles. Cycle distance (**A**), output power (**B**), effective output power (**C**) of the double poling cycle in 30-s experiments.

The results of the temporal parameters of ski propulsion are listed in Table 1. Athlete poling with camber angle of 24° had the smallest CT (t = 1.53 ± 0.17 s) and PT (t = 0.73 ± 0.07 s). The results revealed that the abduction angle affects the cycle distance, in detail, the cycle distance with poling camber angle of 24° (1.07 ± 0.12 m, *P* = 0.029) and 30° (1.11 ± 0.13 m, *P* < 0.001) were significantly larger than 0° (1.02 ± 0.14 m) (Fig. 2A).

**Table 1:** Cycle time (CT), poling phase time (PT) and recovery phase time (RT) of the double poling cycle for different poling camber angles.

Poling camber angle	0°	15°	24°	30°
CT (s)	1.57 ± 0.19	1.55 ± 0.16	1.53 ± 0.17	1.56 ± 0.19
PT (s)	0.76 ± 0.10	0.75 ± 0.09	0.73 ± 0.07	0.75 ± 0.12
RT (s)	0.82 ± 0.10	0.80 ± 0.09	0.81 ± 0.10	0.84 ± 0.09

*CT* cycle time, *PT* poling phase time, *RT time* recovery phase time.

Results in Fig. 2B show that output power increased with the increase of poling camber angle at 15° (597.78 ± 150.31 J), 24° (610.94 ± 158.96 J,* P* = 0.011) and 30° (629.10 ± 168.78 J, *P* < 0.001) compared with 0° (590.65 ± 148.95 J). And result show that effective output power decreased with the increase of poling camber angle at 15° (577.41 ± 145.18 J), 24° (558.12 ± 145.22 J,* P* = 0.011) and 30° (544.82 ± 146.17 J, *P* < 0.001) compared with 0° (590.65 ± 148.95 J).

### Maximum range of motion and joint moment

**Figure 3:**
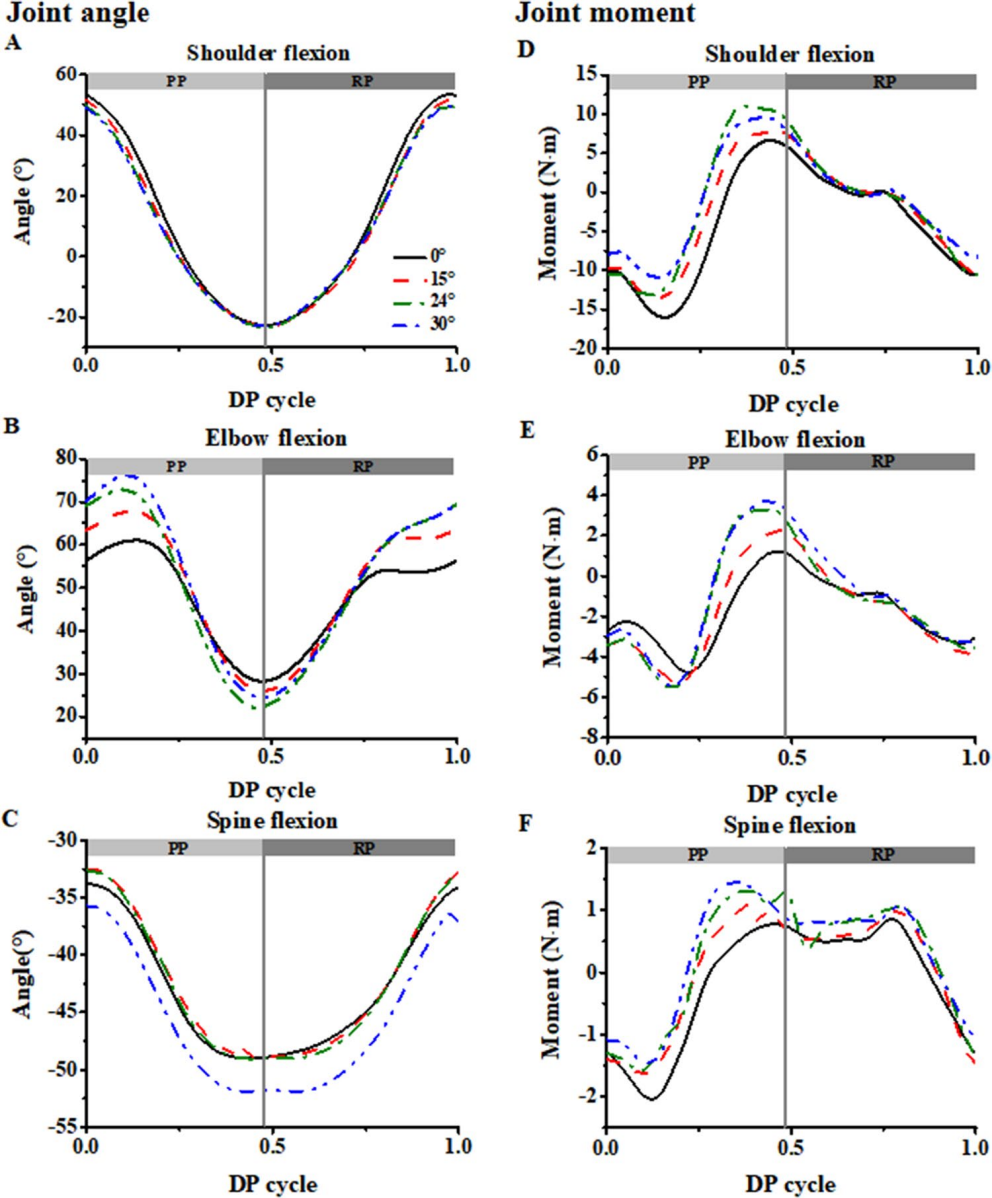
Joint angle and joint moment of shoulder, elbow and spine during the double poling (DP) cycle in cross-country sit-skiing. (**A–C**) Joint angles of shoulder flexion (**A**), elbow flexion (**B**), and spine flexion (**C**). (**D–F**) Joint moment of shoulder flexion (**D**), elbow flexion (**E**), and spine flexion (**F**). All angles are measured in degrees and are presented for 100% double poling (DP) cycle.

Joint kinematics showed that elbow and spine flexion were affected by the poling camber angle during the DP, while shoulder flexion was not (Fig. 3A–C). When athlete performed DP with poling camber angle, the elbow flexed more at the start of the poling phase (Fig. 3B), and the range of motion (ROM) of elbow and spine flexion also increased. Specifically, elbow ROM of poling camber angle at 24° and 30° were significantly higher than the other poling camber angle (0° vs 15°, *P* < 0.001, 0° vs 24°, *P* < 0.001, 0° vs 30°, *P* < 0.001, 15° vs 24°, *P* = 0.032, 15° vs 30°, *P* = 0.002). The ROM of spine flexion was increased with the poling camber angle (0° vs 24°, *P* < 0.001, 0° vs 30°, *P* < 0.001, 15° vs 24°, *P* = 0.037, 15° vs 30°, *P* < 0.001, 24° vs 30°, *P* = 0.021). ROM of pole angle was also increased when athlete performed DP with poling camber angle (0° vs 24°, *P* = 0.025, 0° vs 30°, *P* = 0.013, 15° vs 24°, *P* = 0.026) (Table 2).

**Table 2:** Kinematic data over the double poling cycle for different poling camber angles.

ROM (°)	0°	15°	24°	30°
Shoulder flexion	77.02 ± 18.92	75.84 ± 20.13	75.33 ± 18.88	77.84 ± 20.16
Elbow extension	35.40 ± 10.36	45.34 ± 10.79**	53.89 ± 19.51**^†^	55.40 ± 18.41**^††^
Spine flexion	16.45 ± 7.33	17.95 ± 7.84	19.23 ± 6.31**^†^	21.61 ± 7.27**^††#^
Pole angle	31.90 ± 4.70	32.47 ± 5.37	34.03 ± 4.59*^†^	34.07 ± 5.36*

*ROM* range of motion.

*Represents a significant difference compared to 0°.

^†^Represents a significant difference compared to 15°.

^#^Represents a significant difference compared to 24°.

*^,†,#^p < 0.05, **^,††,##^p < 0.01.

Joint kinetic results revealed that the peak flexion moments of shoulder and elbow occurred at the later stage of the PP (Fig. 3D,E). The peak flexion moments of shoulder (0° vs 15°, *P* = 0.003, 0° vs 24°, *P* = 0.009, 0° vs 30°, *P* = 0.01), elbow (0° vs 15°, *P* = 0.007, 0° vs 24°, *P* = 0.001, 0° vs 30°, *P* = 0.007), and spine (0° vs 30°, *P* = 0.002, 15° vs 30°, *P* = 0.034) increased with the poling camber angle increased when athlete performed DP. The peak flexion moments were not significant changed when the poling camber angle increased from 24° to 30° for both shoulder and elbow joints (Fig. 3D–F, Table S1).

### Muscle activation and muscle force

Average muscle activity (aEMG) and the muscle activity peak (EMGpeak) data were reported as mean ± SD in the Table 3. Both aEMG and EMGpeak values of shoulder joint muscles increased when poling camber angle increased. Specifically, for poling camber angle of 24° or 30°, aEMG value of the Antdelt (0° vs 24°, *P* = 0.033, 15° vs 24°, *P* = 0.025), Middelt (0° vs 24°, *P* = 0.021, 0° vs 30°, *P* = 0.044), Postdelt (0° vs 24°, *P* = 0.03, 0° vs 30°, *P* = 0.034), Bic (0° vs 30°, *P* = 0.016), Tric (0° vs 30°, *P* = 0.049) and Infra (0° vs 30°, *P* = 0.049) increased significantly, and EMGpeak value of Antdelt (0° vs 24°, *P* = 0.028), Middelt (0° vs 24°, *P* = 0.04, 0° vs 30°, *P* = 0.044), Postdelt (0° vs 30°, *P* = 0.023), Bic (0° vs 24°, *P* = 0.04, 0° vs 30°, *P* = 0.045) and Tric (0° vs 24°, *P* = 0.048, 0° vs 30°, *P* = 0.043) increased significantly.

**Table 3:** Average muscle activity (aEMG) and the muscle activity peak (EMGpeak) data of six muscles for different poling camber angles.

	Muscle	0°	15°	24°	30°
aEMG (μV)	Antdelt	9.40 ± 1.52	9.65 ± 2.53	15.81 ± 4.85*^†^	20.05 ± 10.88
	Middelt	12.73 ± 1.67	20.41 ± 6.50*	29.32 ± 11.08*	46.21 ± 26.71*
	Postdelt	33.24 ± 8.50	50.14 ± 9.37	74.02 ± 24.71*	100.84 ± 40.77*^†^
	Bic	12.39 ± 2.39	13.42 ± 1.93*	17.97 ± 4.76	20.39 ± 4.48*^†^
	Tric	13.41 ± 6.22	15.99 ± 8.25	20.78 ± 12.93	24.69 ± 15.06*^#^
	Infra	19.36 ± 4.40	18.60 ± 2.21	24.17 ± 2.97*^†^	28.21 ± 7.09^†^
EMGpeak (μV)	Antdelt	24.48 ± 6.03	22.57 ± 9.03	31.37 ± 7.14*	52.19 ± 32.44
	Middelt	24.09 ± 6.22	55.40 ± 9.88	88.86 ± 47.98*	163.66 ± 107.96*^†^
	Postdelt	101.39 ± 45.96	193.82 ± 57.38	299.55 ± 154.96	384.07 ± 141.96*
	Bic	29.53 ± 10.19	24.34 ± 2.14	38.36 ± 12.79*	43.72 ± 15.34*
	Tric	43.05 ± 27.11	61.09 ± 37.41	80.92 ± 56.50*	106.19 ± 75.14*
	Infra	35.36 ± 17.47	48.58 ± 7.08	57.20 ± 10.75	81.69 ± 34.55

*aEMG* average muscle activity, *EMGpeak* muscle activity peak, *Antdelt* anterior deltoid, *Middelt* middle deltoid, *Postdelt* posterior deltoid, *Bic* biceps brachii, *Tric* triceps brachii, *Infra* infraspinatus.

*Represents a significant difference compared to 0°.

^†^Represents a significant difference compared to 15°.

^#^Represents a significant difference compared to 24°.

*^,†,#^p < 0.05.

**Figure 4:**
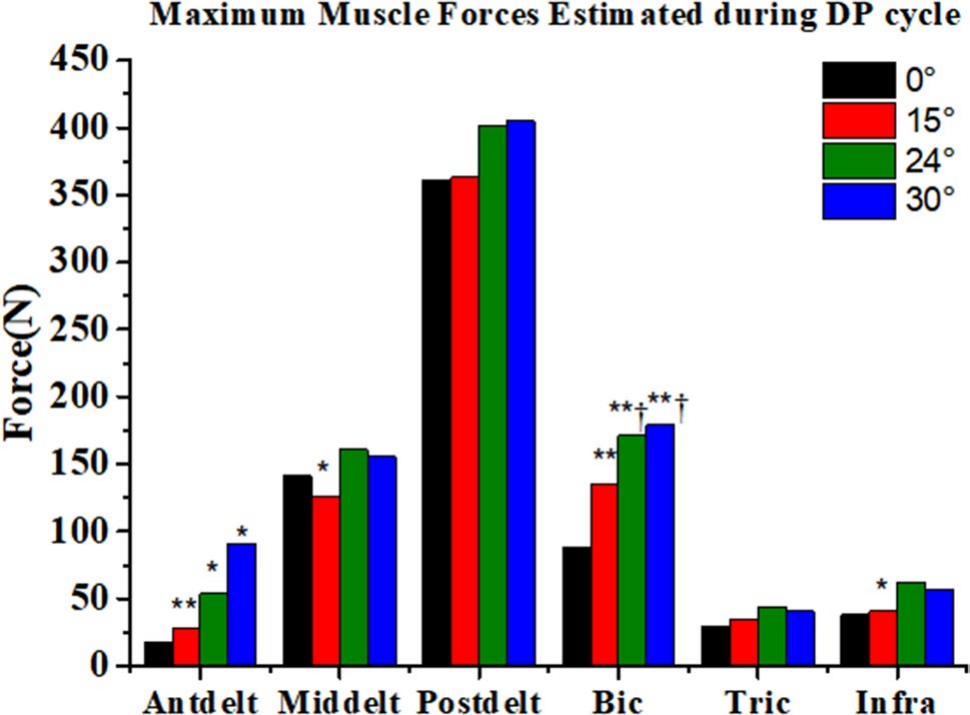
Maximum muscle force estimated during the double poling (DP) cycle. *Represents a significant difference compared to 0°; ^†^represents a significant difference compared to 15°; ^#^represents a significant difference compared to 24°. *^,†,#^p < 0.05, **^,††,##^p < 0.01. Anterior deltoid (Antdelt), Middle deltoid (Middelt), Posterior deltoid (Postdelt), Biceps brachii (Bic), Triceps brachii (Tric), and Infraspinatus (Infra).

SO was used to calculate the muscle forces during the DP, and the maximum muscle forces during the DP process are displayed in Fig. 4. Postdelt, Bic, and Middelt generated the greatest average forces over the six DP cycles with or without the poling camber angle. In addition, maximum muscle forces of Antdelt (0° vs 15°, *P* = 0.005, 0° vs 24°, *P* = 0.023, 0° vs 30°, *P* = 0.046) and Bic (0° vs 15°, *P* = 0.003, 0° vs 24°, *P* = 0.001, 0° vs 30°, *P* < 0.001, 15° vs 24°, *P* = 0.04, 15° vs 30°, *P* = 0.031) significantly increased with the increase of poling camber angles.

## Discussion

To the best of our knowledge, this is the first study to analyze the effect of poling camber angle on the biomechanics of cross-country sit-skiing. We found that the output power increased when poling with the camber angle, as the temporal-spatial parameters such as CT and PT were decreased and cycle distance were increased. Joint flexion of ROM and joint moment of shoulder and elbow were also influenced by the poling camber angle. In addition, Antdelt and Bic were found to be sensitive to the poling camber angle. These findings add to an emerging body of literature dissecting how DP technical skills contributes to the cross-country sit-ski.

The output power analysis of DP process (Fig. 2B) revealed that it increases along with poling camber angle. These findings are consistent with a previous study on wheelchair users^23^, that is, 14 well trained wheelchair court sport athletes drove the wheelchair on a motorized treadmill in four standardized camber conditions (15°, 18°, 20°, and 24°). Their results showed that larger camber angle corresponding to larger output power, i.e. 24° (24.3 ± 5.4 W) vs 15° (20.3 ± 4.0 W, P = 0.004, 24° vs 18° (21.3 ± 4.4 W, P = 0.006), 20° (23.3 ± 5.3 W) vs 15° (P = 0.013)., We believe that pushing the wheelchair with camber angle is similar with the condition of DP with poling camber angle.

The temporal–spatial parameters show that when athletes pole with camber angle, they pole faster (smaller PT and CT) and longer (longer cycle distance). The increase of cycle distance may attribute to the increase of elbow ROM, and the ROM of elbow and spine joint was increased with the poling camber angle. A previous study produced by Ohlsson et al.^16^ underscores that power generation predominantly occurs in the trunk, implying a potential amplification in trunk power. As when athlete pole with camber angle, the elbow get more flexed and extended at the start and end of the poling phase respectively (Fig. 3B). During the PP the shoulder joint exhibits a flexion–extension pattern similar with the elbow^31^. To flex more at the start of the poling phase is easy, but the upper limb did not output power at RP. During the RP, the athlete tries to lift the pole until they support it again. To extend more at the end of the poling phase is hard, as shoulder and elbow have to export larger extension moment (Fig. 3D,E), this is because the moment arm (both shoulder and elbow joint) increased as the elbow extended. In addition, as the upper limb swing more backward, the 3-dimensional pole force has a greater component along the forward direction, which makes the poling more efficient^12^. This is consistent with the output power results in Fig. 2B.

The output power increased when poling with shoulder abducted, and naturally the muscle activation level and muscle forces increased, especially for the deltoids and the Bic (Fig. 4). The deltoids are the stabilizing muscles of shoulder joint^32^, and generate the largest forces during the PP (Fig. 4). As the functional muscle of shoulder abduction, the maximum muscle forces of Antdelt significantly increased when poling with shoulder abduction. In addition, the increase of Bic muscle force may be due to the increase of elbow flexion during the RP, as the RT decreased when poling with the poling camber angle.

Although the results showed that the larger the poling camber angle, the larger the of output power, the poling camber angle not be too large. As we find that at the later stage of the poling cycle, there is a positive correlation in the late stage of DP cycle, the shoulder abduciton angle is about 33° when the camber angel are larger than 30° (Fig. S3). When the shoulder abduction angle is larger than 30°, the stability of the shoulder joint will be reduced and leads to a significant risk of shoulder injury^33^. Meanwhile, the effective output power decreased with a larger camber angel, and a larger shoulder abduction angle at the later stage of the poling cycle (Fig. 2). A larger camber angle decreased the pole angle, particularly at the start of the PP (Fig. S1), which means that the vertical component of pole force is increased. This can be beneficial to the uphill terrain that larger vertical component of the pole force will counteract greater gravity force.

In should be noted that this study has a few limitations. Professional athletes with lower limb impairments were not recruited for this research. For non-disabled athletes, securing the lower legs to the seat may restrict knee movement, potentially impacting the engagement of leg muscles and influencing the flexion angle of the spine. The simulation in the laboratory can not completely simulate the real competition in the snow, in addition, the researchers also did not consider longer testing of DP on the ergometer, which could make the testing more similar to the real situation of a cross-country sit-skiing race.

## Conclusion

The current study revealed that it is beneficial to increase cycle distance and poling speed as the angle of abduction increases. And poling with camber angle increased the output power but decreased the efficiency. These results means the optimal poling camber angle may depend on the terrain or the skiing speed. In detail, cross-country sit-skiers would be recommended to pole with a larger poling camber angle at the start up. Poling with shoulder abducted is beneficial for the muscle power generation, and with the larger vertical component of the pole force counteract with the gravity force, it’s easier to drive the sled at an uphill terrain. And at the flat terrain, the athletes were recommended to pole with a smaller poling camber angle, that the effective output power is larger.

### Supplementary Information

Supplementary Information.

## Data Availability

The datasets used and/or analysed during the current study available from the corresponding author on reasonable request.
