# Supplementary material for: Effects of poling camber angle on the biomechanics of cross-country sit-skiing
Source: Sci Rep. 2023 Nov 28;13:20893. doi: 10.1038/s41598-023-48359-z (PMC10684654; doi:10.1038/s41598-023-48359-z)
Supplement: Supplementary file 1 — Supplementary Information. [file 41598_2023_48359_MOESM1_ESM.docx]

# Supplementary Materials

**Effects of poling camber angle on the biomechanics of cross-country sit-skiing**

**Yuan Tian^1^, Xue Chen^2^, Yujie Liu^1^, Gang Sun^1^, Zhixiong Zhou^3^,**

**Chenglin Liu^1^*, Bo Huo^1^***

^1^Sports Biomechanics Center, Sports Artificial Intelligence Institute, Capital University of Physical Education and Sports, Beijing 100191, P. R. China.

^2^Biomechanics Lab, Department of Mechanics, School of Aerospace Engineering, Beijing Institute of Technology, No. 5 South Zhongguancun Street, Beijing 100081, P. R. China.

^3^School of Physical Education and Coaching Science, Capital University of Physical Education and Sports, Beijing 100191, P. R. China.

***Corresponding authors:**

Dr. Chenglin Liu, Sports Biomechanics Center, Sports Artificial Intelligence Institute, Capital University of Physical Education and Sports, No. 11 North Third Ring Road West, Beijing 100191, P. R. China, E-mail: [liuchenglin@cupes.edu.cn](mailto:liuchenglin@cupes.edu.cn)

Dr. Bo Huo, Sports Biomechanics Center, Sports Artificial Intelligence Institute, Capital University of Physical Education and Sports, No. 11 North Third Ring Road West, Beijing 100191, P. R. China, E-mail: [huobo@cupes.edu.cn](mailto:liuchenglin@cupes.edu.cn)

## Figure S1. Pole angle and pole force during the double poling (DP) cycle in cross-country sit-skiing. Pole angle (A) and pole force (B).


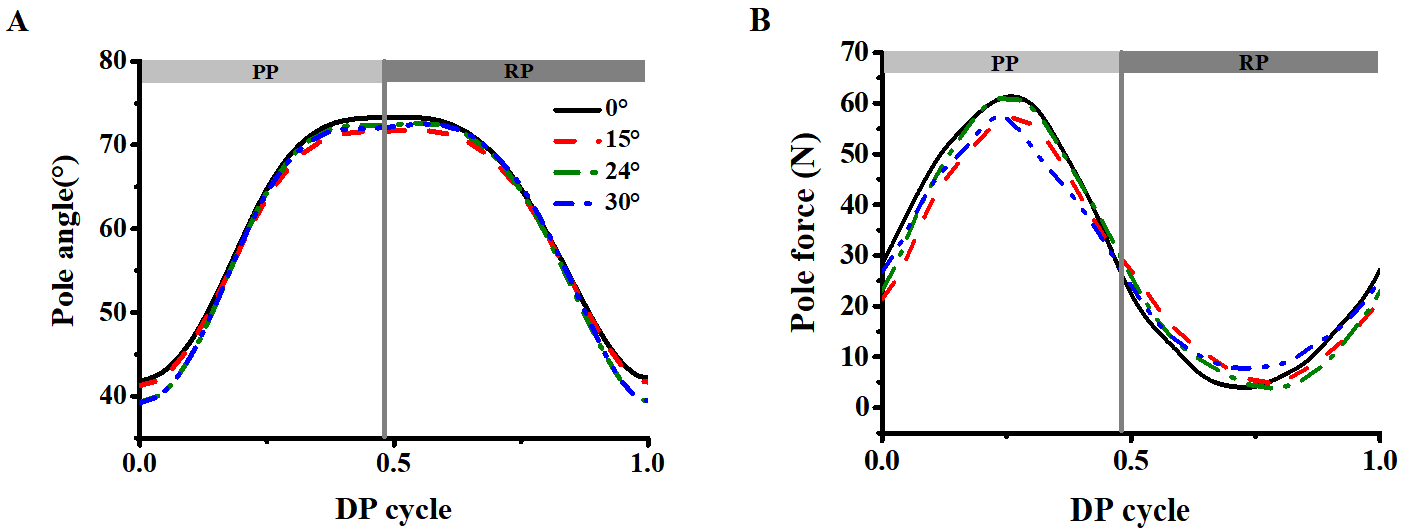


## Figure S2. Force components sikiing.


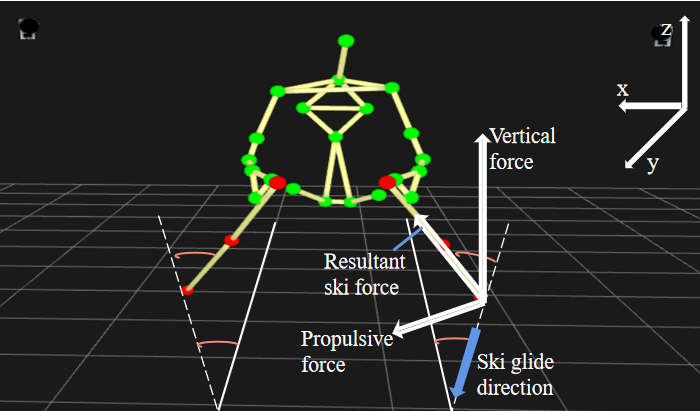


**Figure S3.** Shoulder abduction within the DP cycle under different poling camber angle.


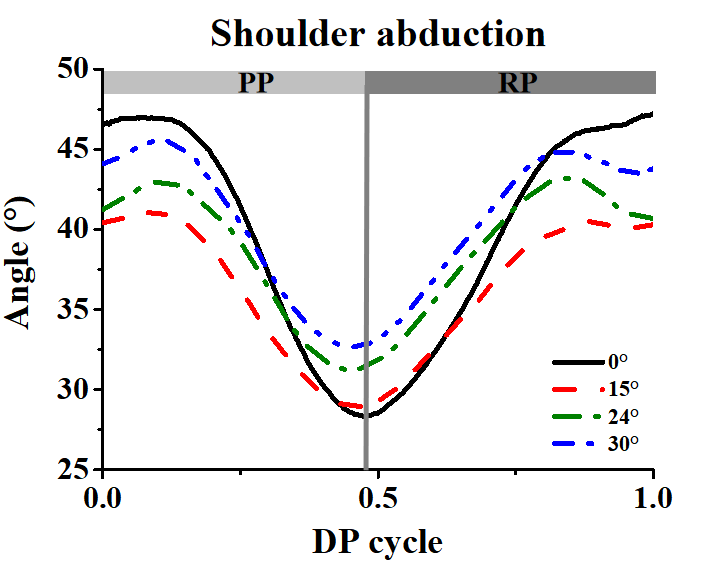


## Table S1. Kinetic data over the double poling cycle for different poling camber angles.

| Peak | 0° | 15° | 24° | 30° |
| --- | --- | --- | --- | --- |
| Shoulder flexion moment (N∙m) | 7.03±3.69 | 10.38±5.06^**^ | 13.25±6.47^**^ | 13.66±6.63^**^ |
| Elbow flexion moment (N∙m) | 1.76±1.87 | 3.15±2.46^**^ | 4.67±2.88^**^ | 5.37±3.60^**†^ |
| Trunk moment (N∙m) | 1.27±0.37 | 1.62±0.67 | 2.25±2.14 | 2.05±0.77^*†^ |
| Pole force (N) | 70.96±15.93 | 67.51±17.98 | 72.79±17.19 | 69.02±16.46 |

**Note:** * represents a significant difference compared to 0°, † represents a significant difference compared to 15°; # represents a significant difference compared to 24°. *, †, #, p<0.05, **, ††, ##, p<0.01.
